# Supplementary material for: LIM Protein Ajuba associates with the RPA complex through direct cell cycle-dependent interaction with the RPA70 subunit
Source: Sci Rep. 2018 Jun 22;8:9536. doi: 10.1038/s41598-018-27919-8 (PMC6015067; doi:10.1038/s41598-018-27919-8)
Supplement: Supplementary file 1 — Supplementary Information [file 41598_2018_27919_MOESM1_ESM.pdf]

Supplementary information

LIM Protein Ajuba associates with the RPA complex through direct cell cycle-dependent interaction with the RPA70 subunit

Sandy Fowler, Pascal Maguin, Sampada Kalan and Diego Loayza

Supplementary Figures:

Figure S1. Ajuba-RPA70 interaction is reduced during replication stress. IP-Western from total extracts in unsynchronized HTC75 (left) and IMR90 (right) cells. Cells were untreated and treated with 2mM HU for 24 hours (+2mM HU). The * denotes a non-specific band.

Figure S2. Ajuba protein levels do not change during hydroxyurea treatment. HTC75 cells were treated with 2mM HU for 3, 6, and 24 hours and processed for protein extraction and Western blotting.

Figure S3. Flow cytometry for propidium iodide content of HTC75 cells (see Figure 1C) and IMR90 cells (see Figure S4). Cells were untreated (UT), treated with 2mM HU for 24 hours (24 he HU), and synchronized to S phase by double thymidine block and release for 2 hours (dT 2hr) or 4.5 hours (dT 4hr).

Figure S4. Ajuba-RPA32 interaction is reduced during replication stress but not in S phase cells. IMR90 cells were untreated, treated with 2mM HU for 24 hours (24 hr 2mM HU), or synchronized to S phase by double thymidine block and released for 4.5 hours (double thymidine S phase).

Figure S5. Ajuba-RPA70 nuclear co-localization is increased by Leptomycin B treatment (10uM). A) Co-immunofluorescence of Ajuba and RPA70 in unsynchronized HTC75 and IMR90 cells. Arrowheads point to sites of co-localization B) Quantification of cells that exhibited >3 foci of Ajuba-RPA70 co-localization in untreated and leptomycin B treated HTC75 cells (n=100 cells, on 3 independent experiments).

Figure S6. A) Ajuba displays significant co-localization with PCNA. Co-immunofluorescence of Ajuba and PCNA in unsynchronized and synchronized (double thymidine block and release) HTC75 cells. B) Ajuba displays significant co-localization with BrdU in a subset of BrdU-positive cells. A culture of asynchronous HTC75 cells was pulsed with BrdU for 1 hour and processed for BrdU and Ajuba co-immunofluorescence.

Figure S7. A) Ajuba does not directly interact with POT1. Binding experiment of full-length His-Ajuba and co-translated full-length POT1 followed by autoradiography. B) Binding experiment between co-translated RPA70 OB folds B-C and full-length Ajuba-His followed by autoradiography.

Figure S8. LIM domain 1 of Ajuba and OB fold A of RPA70 mediate the direct interaction between the two proteins. Schematic of Ajuba and RPA70, arrows pointing up represent the positions used to generate truncation mutants.

Figures S9-S13. Full-length blots and scans showed in the main figures. A dashed red box within each blot or scan indicates the approximate areas used to produce the panels shown in the main figures.

Supplementary figures.

Figure S1.

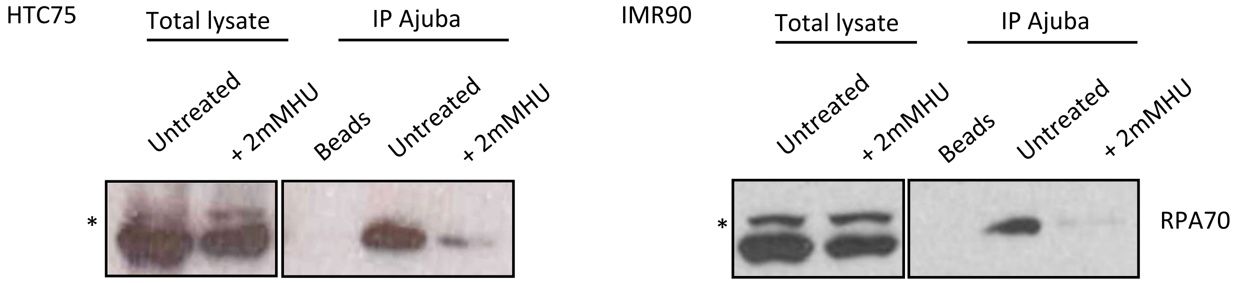

Figure S2.

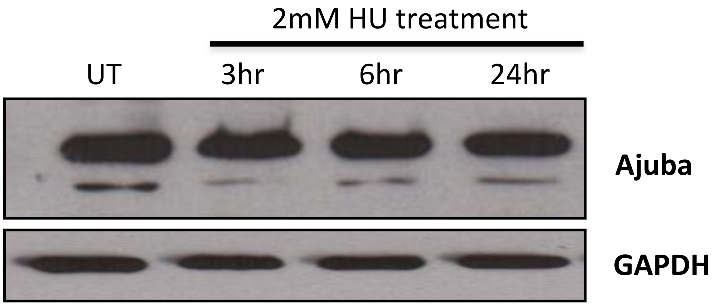

Figure S3.

HTC75	UT	24hr HU	dT 2hr
G1	64%	58.85%	22.1%
S	24%	41.14%	76.7%
G2/M	11%	0%	1.62%

IMR90	UT	24hr HU	dT 4.5hr
G1	63%	55.5%	20%
S	8%	44%	73%
G2/M	29%	0%	4%

Figure S4.

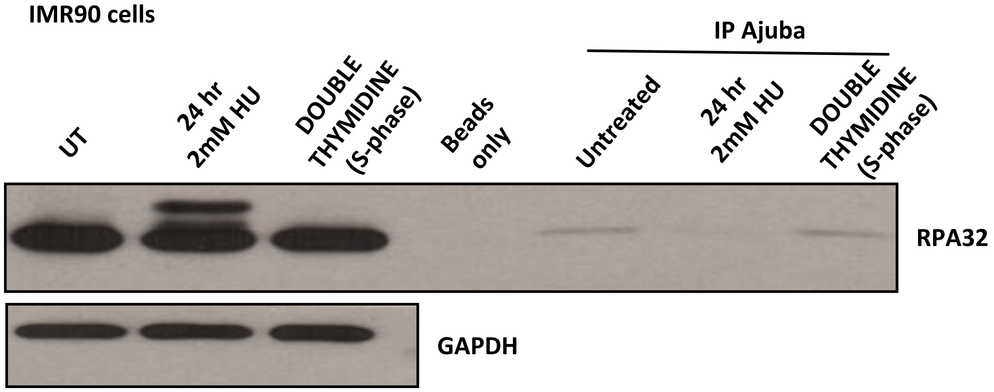

Figure S5.

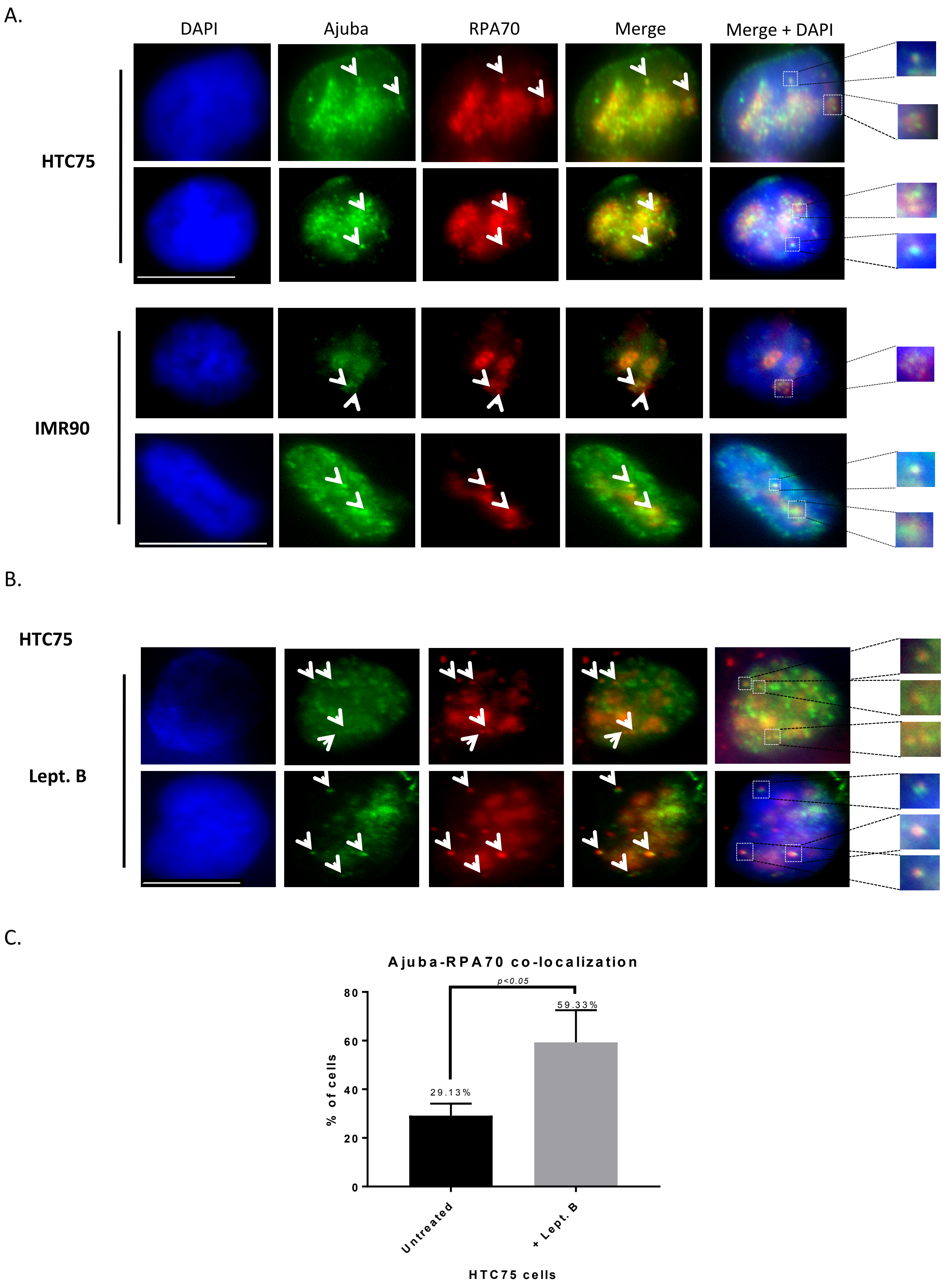
A.
DAPI
Ajuba
RPA70
Merge
Merge + DAPI
HTC75
IMR90
B.
HTC75
Lept. B
C.
Ajuba-RPA70 co-localization
p<0.05
59.33%
29.13%
80
60
40
20
0
% of cells
Untreated
+ Lept. B
HTC75 cells

Figure S6.

A.

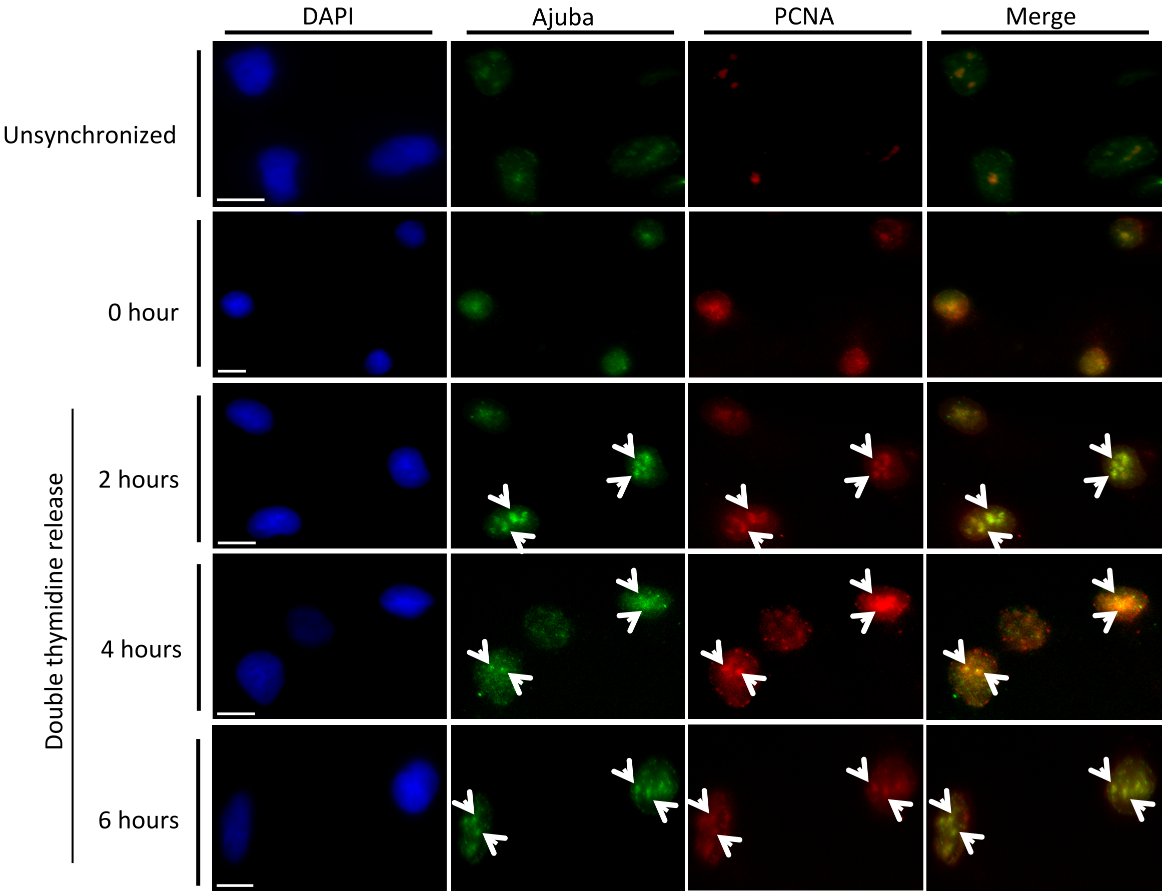
DAPI
Ajuba
PCNA
Merge
Unsynchronized
0 hour
2 hours
4 hours
6 hours
Double thymidine release

B.

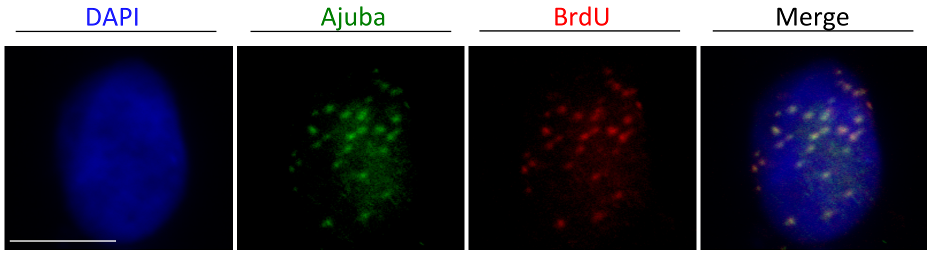
DAPI
Ajuba
BrdU
Merge

Figure S7.

A.

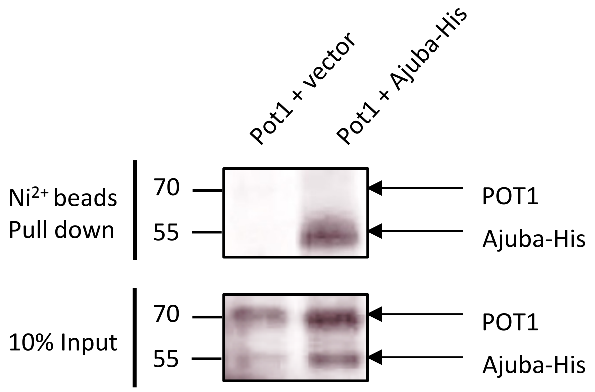
Pot1 + vector
Pot1 + Ajuba-His
Ni^{2+} beads
Pull down
70
55
POT1
Ajuba-His
10% Input
70
55
POT1
Ajuba-His

B.

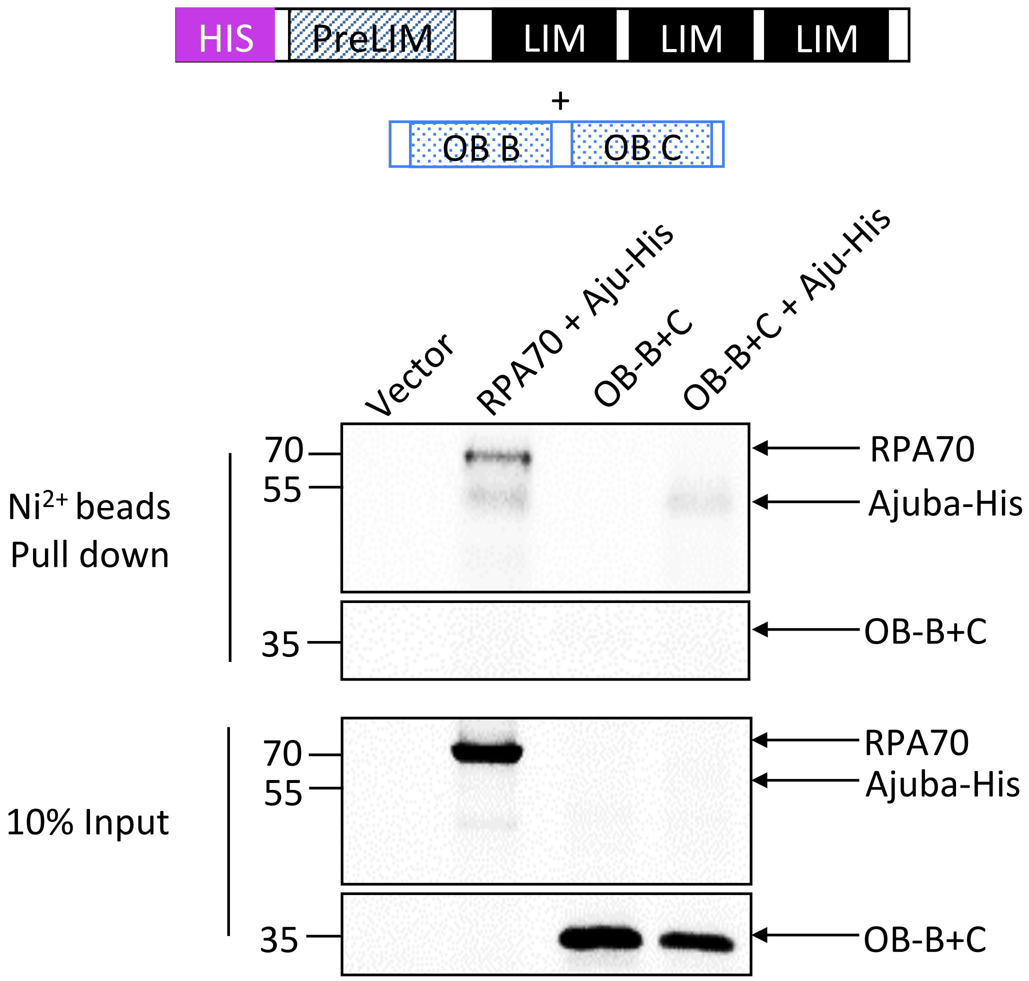
HIS
PreLIM
LIM
LIM
LIM
+
OB B
OB C
Vector
RPA70 + Aju-His
OB-B+C
OB-B+C + Aju-His
Ni^{2+} beads
Pull down
70
55
35
RPA70
Ajuba-His
OB-B+C
10% Input
70
55
35
RPA70
Ajuba-His
OB-B+C

Figure S8.

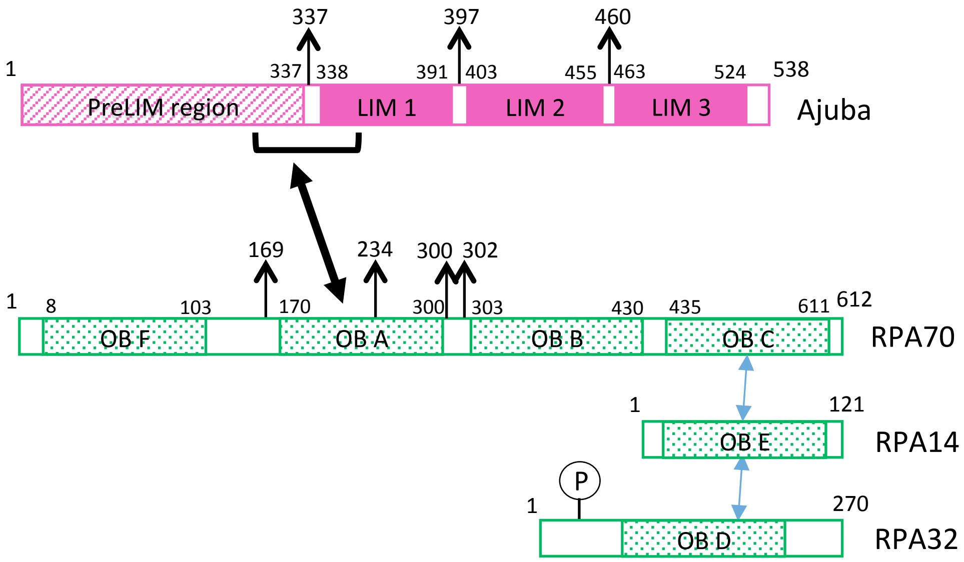

337
397
460
1
337
338
391
403
455
463
524
538
PreLIM region
LIM 1
LIM 2
LIM 3
Ajuba
169
234
300
302
1
8
103
170
300
303
430
435
611
612
OB F
OB A
OB B
OB C
RPA70
1
121
OB E
RPA14
P
1
270
OB D
RPA32

Figure S9.

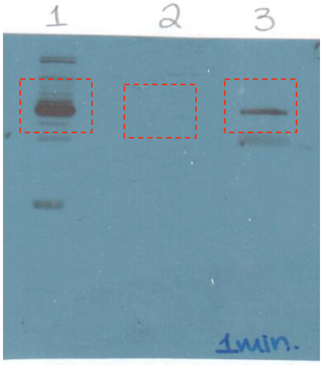

RPA70

Fig. 1A (top)

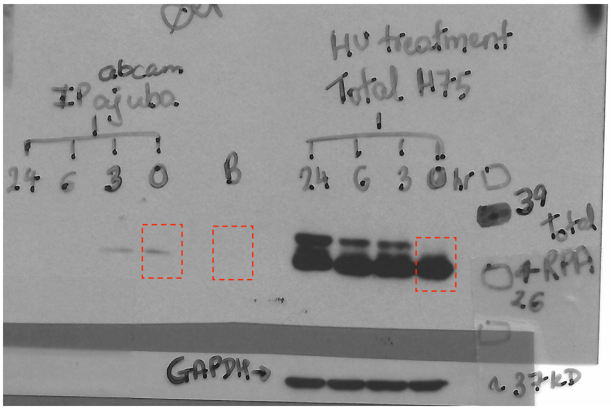

Fig. 1A (bottom)

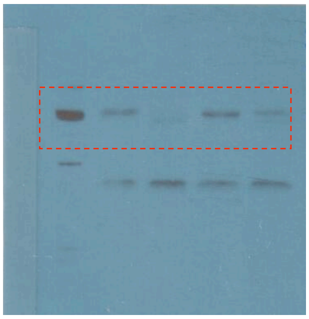

Fig. 1B

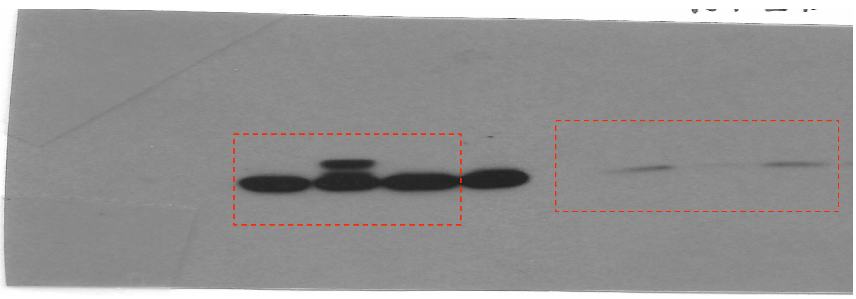

Fig. 1C

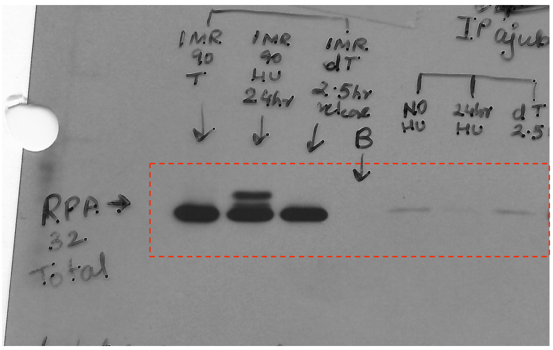

Fig. S2

Figure S10.

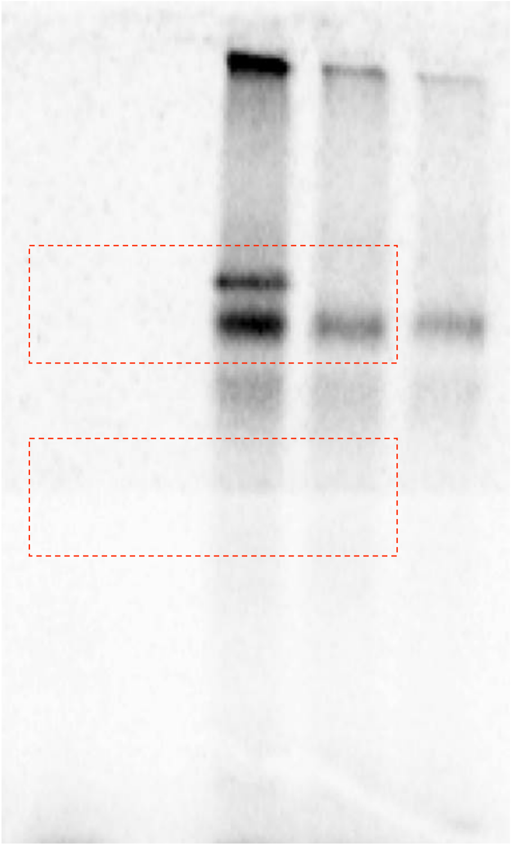

Fig5A (top)

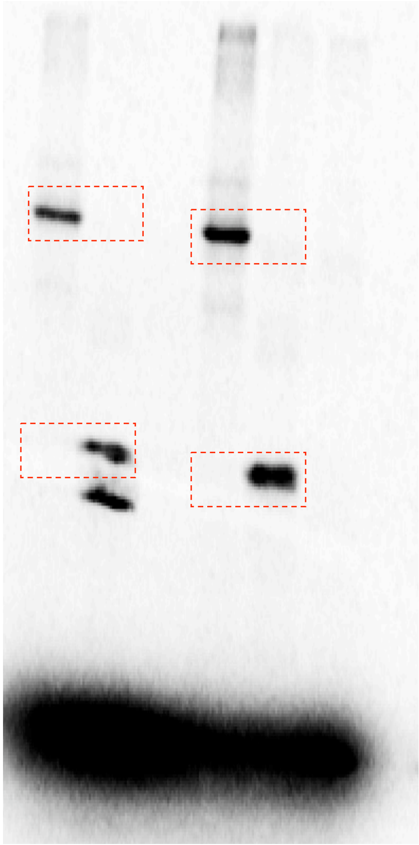

Fig5A (bottom)

Figure S11.

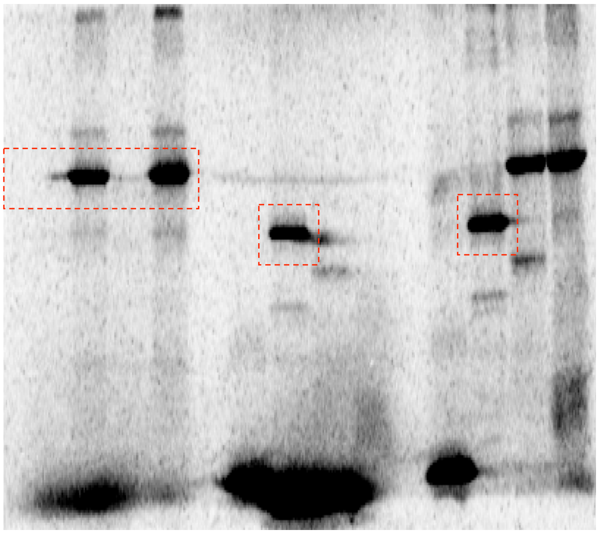

Fig.6A (bottom)

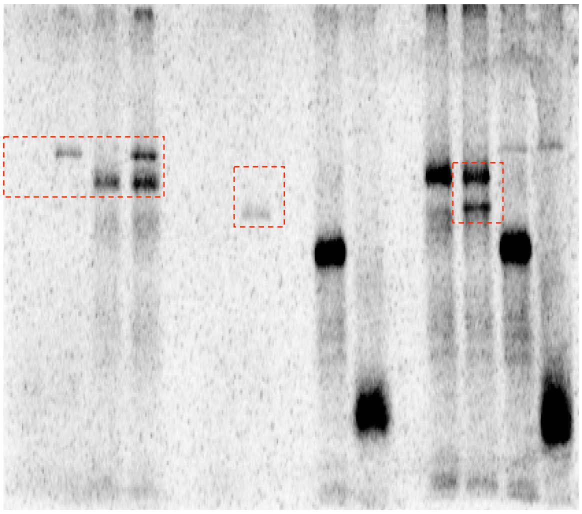

Fig.6A (top)

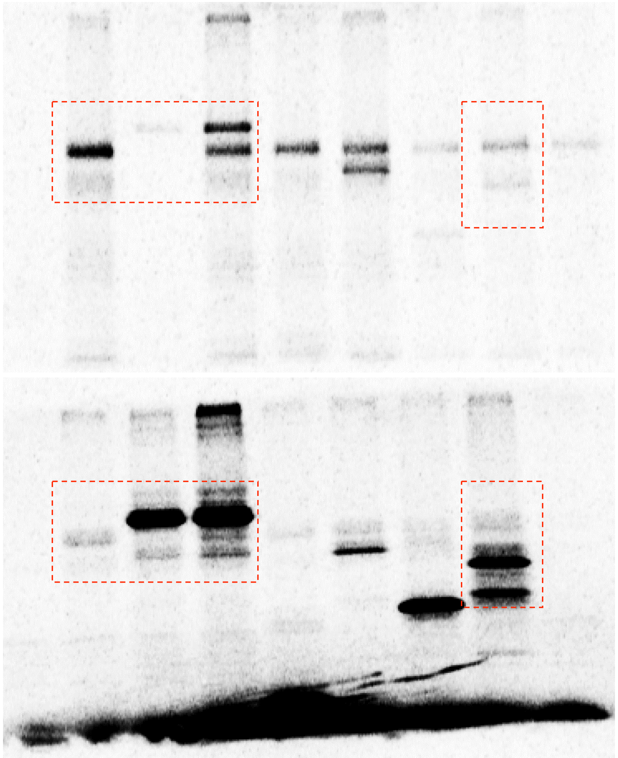

Fig.6B

Figure S12.

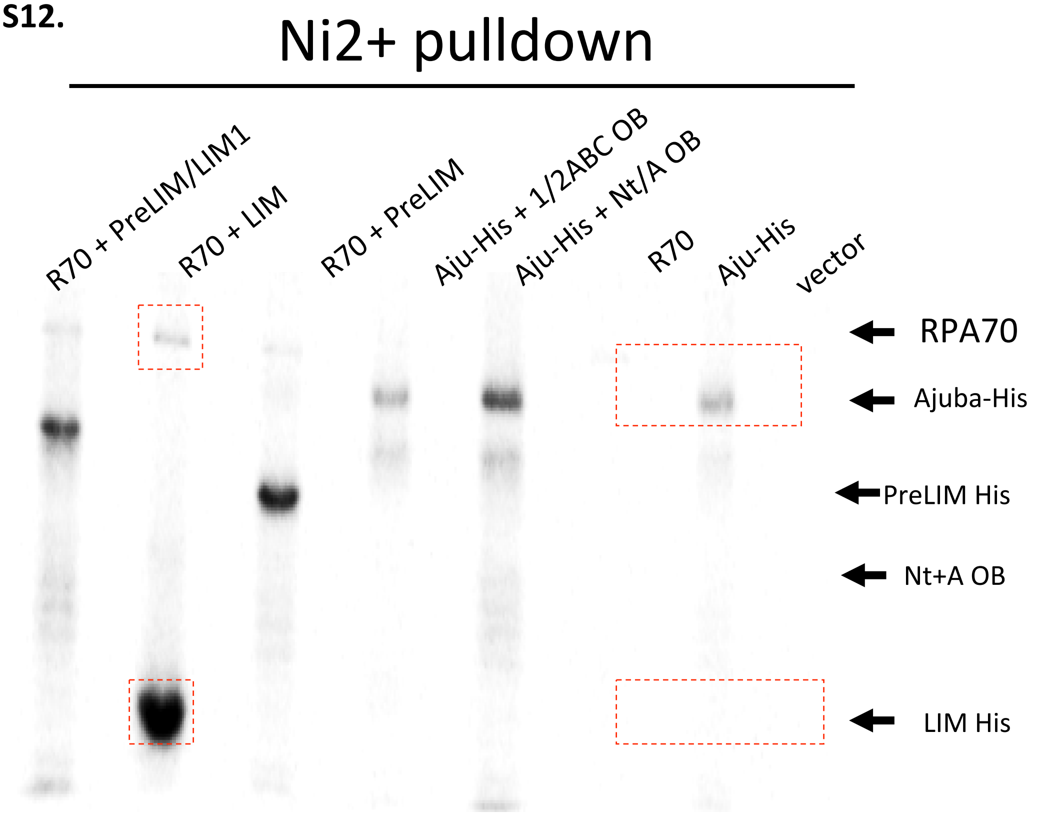

Fig.6C (top)

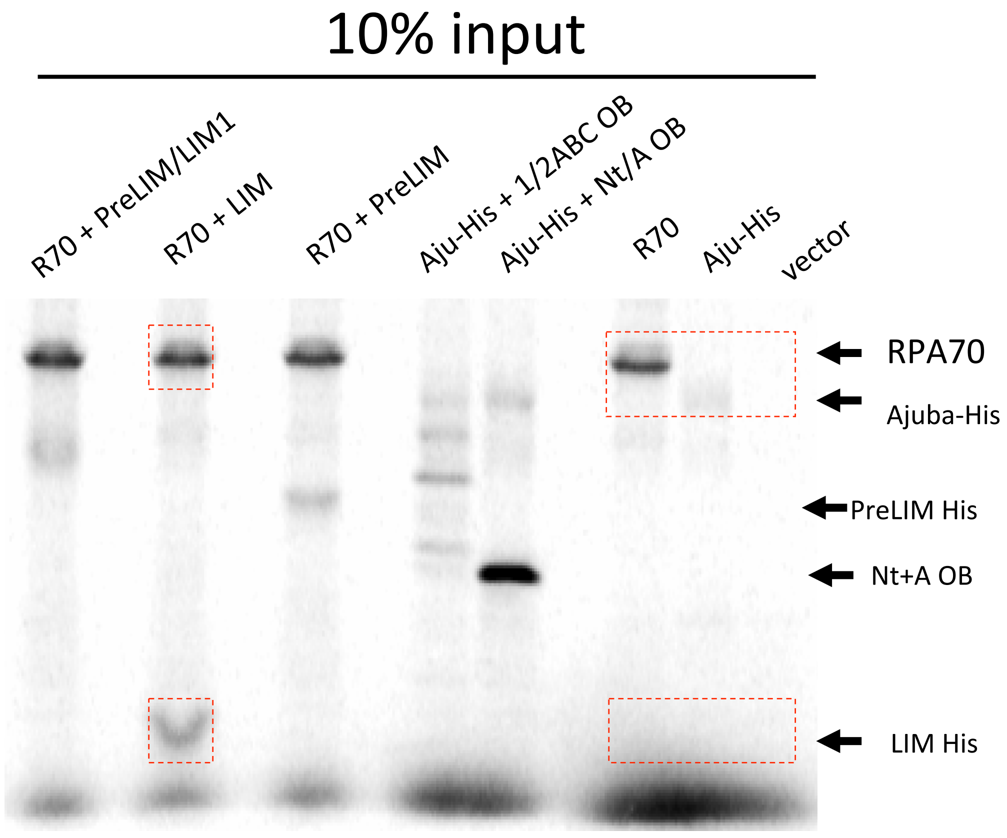

Fig.6C (bottom)

Figure S13.

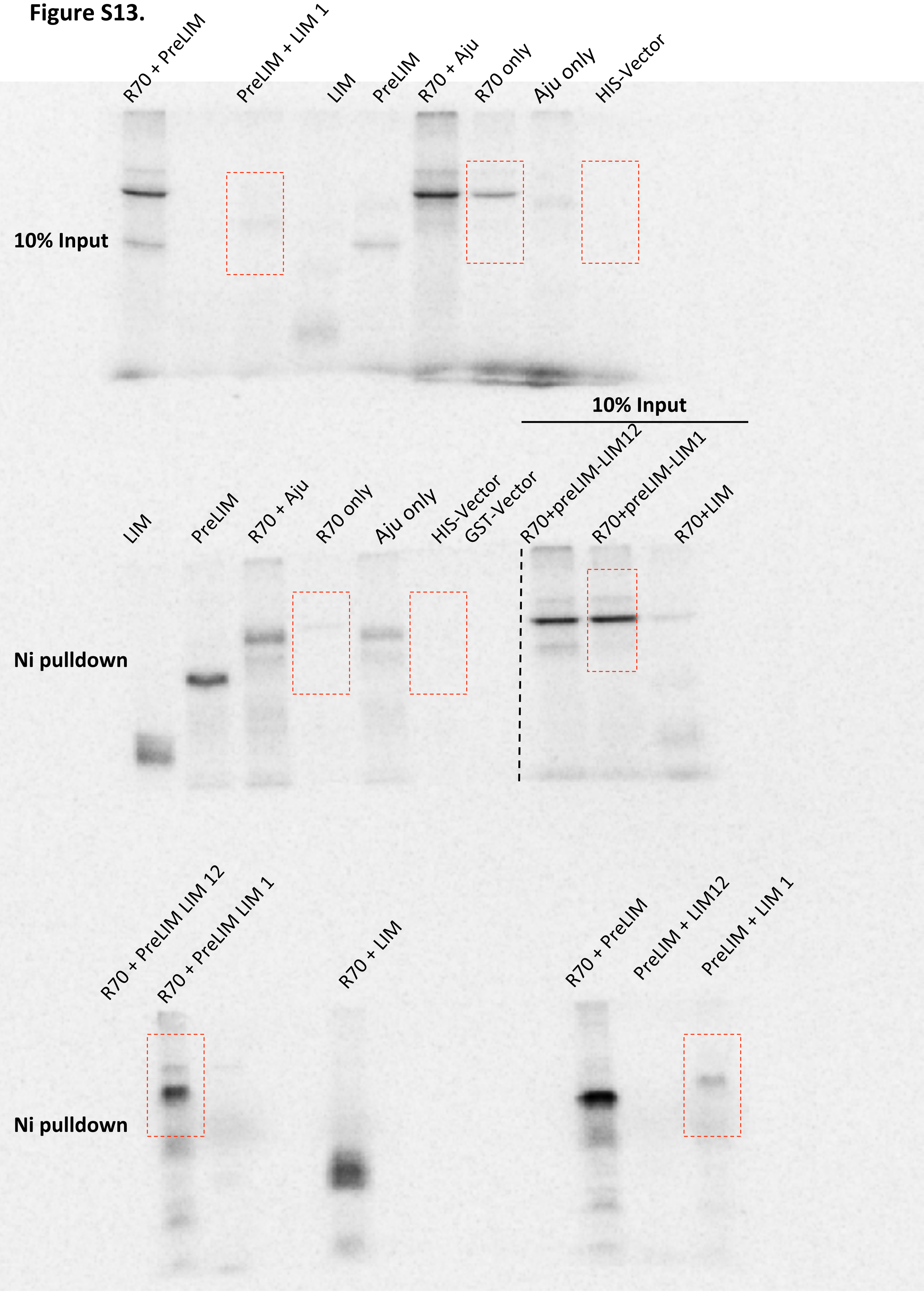

Fig.6D
